# Montelukast and Bilastine Combination Therapy in Allergic Rhinitis: A Narrative Review of Evidence

**DOI:** 10.7759/cureus.113572

**Published:** 2026-07-29

**Authors:** Surinder Jindal, Naveed N Shah, Prachi Jain, Ketaki Patil, Chaitanya Bhargave, Chintan Khandhedia, Suyog Mehta

**Affiliations:** 1 Pulmonary Medicine, Postgraduate Institute of Medical Education and Research, Chandigarh, IND; 2 Respiratory Medicine, Chest Care and Breathe Easy Hospital, Srinagar, IND; 3 Medical Affairs, Sun Pharma Laboratories Limited, Mumbai, IND; 4 Pharmacology, Sun Pharmaceutical Industries Limited, Mumbai, IND; 5 Medical Affairs and Clinical Research, Sun Pharmaceutical Industries Limited, Mumbai, IND; 6 Medical Affairs and Clinical Research, Sun Pharma Laboratories Limited, Mumbai, IND

**Keywords:** allergic rhinitis, bilastine, clinical effects, current gaps, montelukast, odt

## Abstract

Allergic rhinitis (AR) is a chronic inflammatory disorder of the nasal mucosa triggered by immunoglobulin E (IgE)-mediated responses to allergens. The release of histamines and leukotrienes results in nasal irritation, excessive secretions, and frequent sneezing, affecting both children and adults.

This review aims to provide an updated understanding of AR by evaluating the prevalence, treatment approaches, and clinical effects of bilastine and montelukast in AR management. A comprehensive literature search was conducted to identify research papers published between January 2003 and September 2023. Studies assessing the clinical efficacy of montelukast and bilastine, alone or in combination, in AR treatment were reviewed.

Several clinical studies suggest that montelukast and bilastine combination therapy significantly improves daytime and composite nasal symptoms, including itching, sneezing, and rhinorrhea. Evidence indicates that combination therapy is superior to monotherapy with either drug for managing moderate-to-severe persistent AR. Bilastine, a second-generation antihistamine, offers rapid symptom relief with minimal sedation, while montelukast, a leukotriene receptor antagonist, reduces inflammation. Their complementary mechanisms enhance overall efficacy in symptom control.

Although combined bilastine and montelukast therapy is supported by growing clinical evidence, data on their comparative effectiveness against other commercially available AR treatments remain limited. Further research is needed to establish optimal treatment strategies and long-term safety.

## Introduction and background

Allergic rhinitis (AR), commonly known as hay fever, is a prevalent atopic condition characterized by various symptoms, including nasal discharge (rhinorrhea), nasal itching, nasal congestion, and sneezing. This condition is primarily triggered by immunoglobulin E (IgE)-mediated reactions to inhaled allergens [1]. These immune reactions, in turn, aggravate mucosal inflammation, which is mainly initiated by type 2 helper T (Th2) cells [2]. AR has shown a significant rise since the 1990s and imposes a substantial global burden and disability [3-5]. Worldwide, approximately 40% of children and 25% of the adult population are affected by this condition. It has been reported that around 80% of symptoms tend to manifest before the age of 20, peaking between the ages of 20 and 40, and then gradually diminishing over time [6]. According to reported data, the incidence rate of AR among children during the first five years of life is 17.2%, with the highest frequency of diagnosis observed between 24 and 29 months, accounting for 2.5% of cases [7]. In India, the prevalence of AR among adolescents is around 40%, highlighting the considerable number of individuals affected by this condition in the country [8].

The drugs commonly recommended for the management of AR include oral and intranasal antihistamines, oral and intranasal corticosteroids, leukotriene receptor antagonists (LTRAs), anticholinergics, and nasal decongestants [9]. Due to the significant role of histamine in exacerbating allergic responses, antihistamines have been used in clinical practice for more than 70 years to treat allergic conditions, such as urticaria and AR [9,10]. However, the coexistence of AR and asthma is considerably high, with a prevalence rate of up to 80%. Notably, studies have shown that the administration of antihistamines, either alone or in combination with LTRAs, is effective in attenuating asthma symptoms among individuals with seasonal AR [11]. LTRAs such as pranlukast, montelukast, and zafirlukast are drugs of choice for the treatment of asthma and other allergic conditions [12]. Among these, montelukast is the preferred LTRA and the only approved drug for managing AR in pediatric patients [13]. In March 2020, the US FDA issued a safety warning regarding montelukast due to reports of serious neuropsychiatric events, including suicidal thoughts, leading to the addition of a boxed warning in its prescribing information [14]. Combination therapy with oral antihistamines and LTRAs is commonly used to manage persistent or multifactorial AR symptoms, particularly when intranasal corticosteroid sprays are contraindicated or not preferred. Antihistamines alleviate histamine-mediated symptoms but have minimal effect on nasal obstruction or the late-phase inflammatory response driven by mediators such as leukotrienes and cytokines. Cysteinyl leukotrienes (CysLTs) contribute to persistent inflammation by increasing vascular permeability, mucus production, and eosinophilic infiltration. Montelukast, a CysLT1 receptor antagonist, helps reduce nasal obstruction, suppress sneezing and itching, and improve mucociliary clearance. Therefore, combining antihistamines with LTRAs may improve symptom control by targeting multiple inflammatory pathways [15]. The extensive use of antihistamine-LTRA combinations, either as monotherapy or as adjuncts to local corticosteroids, has demonstrated beneficial effects in reducing inflammatory markers [11]. Hence, the combination of potent antihistamines and LTRAs such as bilastine-montelukast may effectively mitigate allergic symptoms by inhibiting key inflammatory mediators, namely, histamine and leukotrienes. This approach may provide protective effects and significantly alleviate associated symptoms.

Intranasal corticosteroids (INCS) and oral H1-antihistamines represent first-line treatments for AR, offering effective symptom control in both intermittent and persistent forms [16,17]. However, limitations such as suboptimal relief of nasal congestion, delayed onset of action with some agents, and incomplete coverage of mixed inflammatory pathways often necessitate adjunctive therapies, such as montelukast-bilastine combinations [18].

While substantial knowledge exists regarding AR, several gaps persist that hinder a comprehensive understanding among medical practitioners. Among these, a key area requiring further investigation is the underlying cause of AR. Although it is well established that the condition results from an overreaction of the immune system to allergens such as pollen or dust mites, the precise mechanisms triggering this response remain unclear. Moreover, there is inadequate information regarding recent advances in effective treatment approaches for the management of AR. Thus, this review paper aims to highlight the pathomechanisms of AR and the role of antihistamines and LTRAs in its clinical management. Additionally, we attempt to address current gaps related to recent advances in the combined therapeutic effects of montelukast and bilastine in AR.

## Review

Materials and methods

An extensive search of studies published in the last two decades was conducted, covering the prevalence, treatment strategies, and combined effects of bilastine and montelukast in the management of AR. Databases searched included PubMed, Google Scholar, MEDLINE, and ScienceDirect, using the keywords “allergic rhinitis”, “bilastine”, “montelukast”, “synergistic effects”, “guidelines”, “management”, and “clinical trials”. The search was limited to studies involving humans and published in English. The search period spanned from January 2003 to September 2023. Proceedings of meetings and case reports were also included. Articles published before the defined search period were used only to provide conceptual background. We also reviewed the reference lists of identified randomized controlled trials (RCTs) to identify additional relevant studies, including unpublished trials or those not captured through electronic searches. Furthermore, ongoing trials were identified through the World Health Organization’s International Clinical Trials Registry Platform (http://www.who.int/ictrp/en/) and ClinicalTrials.gov (www.clinicaltrials.gov).

Objective

The present review provides a comprehensive update on the current knowledge and understanding of AR. It covers various aspects of the condition, including its etiology, clinical manifestations, and prevalence. Additionally, the review analyzes the scientific evidence related to the combined use of montelukast and bilastine fixed-dose combinations (FDCs) as a potential treatment option for AR. A concerted effort has been made to evaluate the safety profiles of montelukast and bilastine, including orally disintegrating tablet (ODT) formulations, as well as their effectiveness in alleviating AR symptoms. Relevant data have been collected and examined to provide an updated understanding of AR. The assessment process involves an in-depth review of current research studies, clinical trials, and observational data to ensure a broad and comprehensive evaluation.

Overview of rhinitis: understanding allergic and non-allergic variations

Rhinitis is one of the most prevalent chronic conditions, characterized by a range of nasal symptoms such as sneezing, pruritus, rhinorrhea, and nasal congestion resulting from inflammation or dysfunction of the nasal mucosa [19]. Other conditions that may present with symptoms similar to rhinitis include primary ciliary dyskinesia syndrome, cerebrospinal fluid rhinorrhea, rhinosinusitis with or without nasal polyps, and pharyngonasal reflux. Individuals with rhinitis often experience significant challenges that adversely affect their quality of life (QOL), including depression, daytime somnolence, fatigue, memory deficits, impaired learning, irritability, and reduced physical and social functioning [20]. It also negatively impacts overall work productivity in approximately 33% of individuals, exceeding the rates observed in chronic obstructive pulmonary disease (COPD) (15%) and asthma (20%) [21]. In addition, 35%-50% of adults with rhinitis experience a moderate impact from nasal allergies in their daily lives [20].

AR and non-allergic rhinitis (NAR) are the two main phenotypes of rhinitis [19]. AR is a chronic inflammatory condition characterized by both nasal and non-nasal symptoms [22]. These symptoms may persist for several hours following exposure to allergens that trigger inflammation of the nasal mucosal lining [6]. Common nasal symptoms in AR include nasal blockage, sneezing, itching, and anterior or posterior rhinorrhea (runny nose) [22], while non-nasal symptoms include ocular manifestations, particularly allergic rhinoconjunctivitis, characterized by tearing, redness, and itching of the eyes [4]. In contrast, patients with NAR experience rhinitis symptoms but do not show clinical evidence of infection (e.g., discolored nasal secretions) or IgE-mediated allergic inflammation, such as a positive skin prick test or detectable allergen-specific IgE in the blood [19,23].

Epidemiological aspects of AR versus NAR

The escalating incidence of AR has transformed it into a global health concern, affecting approximately 25% of the population worldwide. This condition impacts one in every six individuals, leading to significant morbidity, increased healthcare costs, and reduced productivity. The prevalence of AR among children is notably high, making it one of the most common chronic conditions in the pediatric population [24], while NAR affects approximately 1%-50% of the total pediatric population and accounts for 16%-89% of chronic rhinitis cases [25]. The third phase of the International Study of Asthma and Allergies in Childhood (ISAAC) reported a 7% prevalence of AR among children aged 6-7 years and 13-14 years in India. However, some regions have reported increasing rates of up to 10%-20% [26]. A study conducted among children with chronic rhinitis found that the majority of cases were diagnosed with AR (75.9%), while the remaining 24.1% were classified as NAR [27]. Another cross-sectional analysis reported a higher proportion of moderate-to-severe AR (40%) compared to NAR (24%). It also indicated 31% of cases as persistent AR and 25% as NAR [28]. Moreover, numerous epidemiological studies have highlighted a significant decline in the QOL of individuals with AR [3,29]. In addition, AR symptoms can substantially impair health-related QOL (HRQOL) and increase the risk of various comorbidities in both adults and children, including recurrent respiratory infections, sinusitis, asthma, orthodontic malocclusions, and otitis media. These conditions further contribute to poor HRQOL outcomes [30]. More than 30% of patients experience severe AR symptoms that may lead to significant impairment and, in rare cases, potentially life-threatening complications such as anaphylaxis and asthma [31]. In contrast, individuals with NAR may also experience considerable QOL impairment, partly due to the limited availability of evidence-based treatment options [32]. While substantial evidence exists regarding QOL impairment in AR patients [30,31], the extent of health-related QOL impairment in NAR patients remains insufficiently studied [32].

Pathology of AR

**Figure 1:**
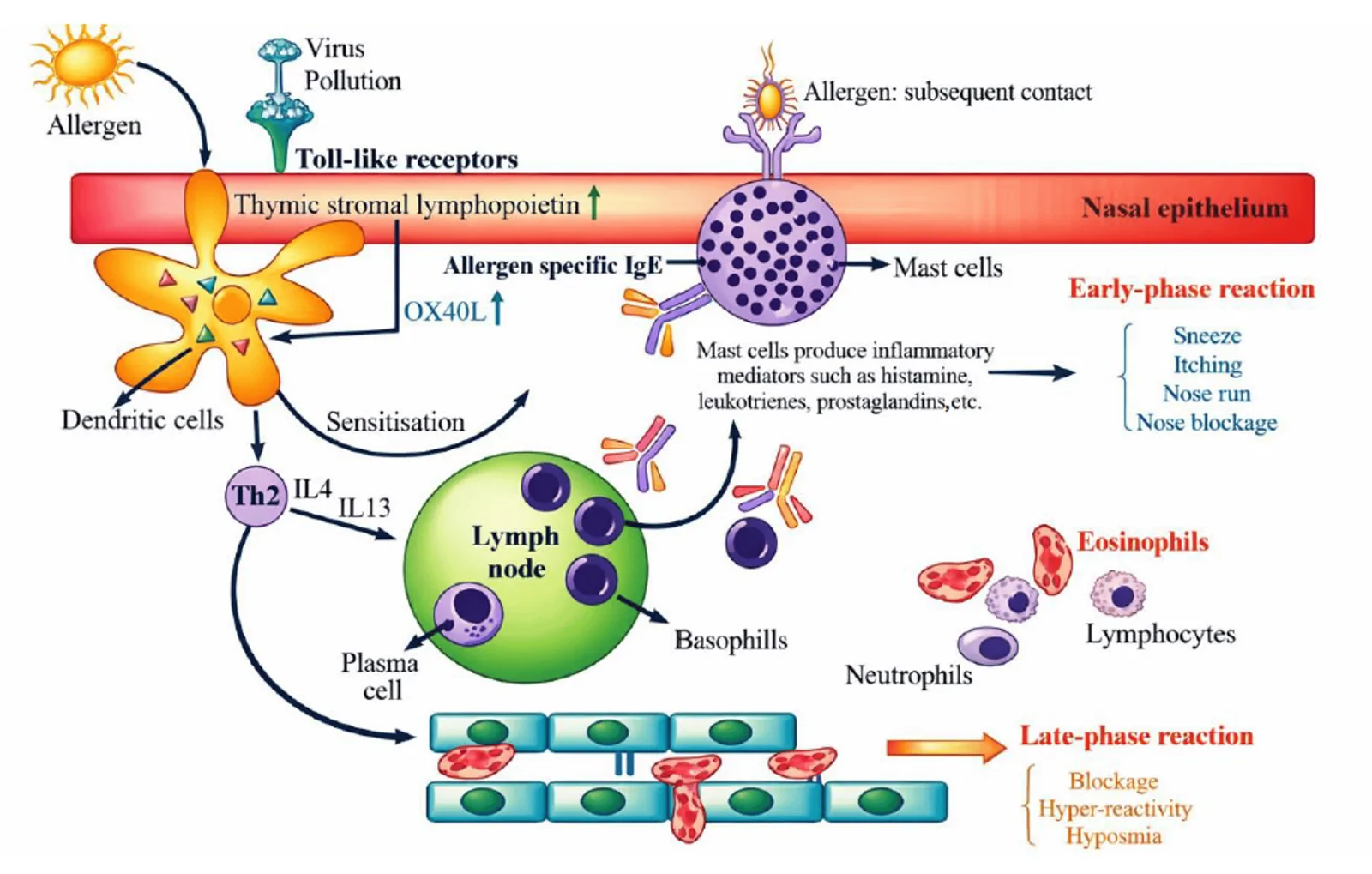
Pathophysiology of allergic rhinitis (AR) This image was created using Microsoft PowerPoint (Microsoft Corp., Redmond, WA, USA). Source: [2,10,33].

Following exposure to allergens, patients with AR exhibit two distinct patterns of allergic response, known as early-phase and late-phase reactions, based on their timing. During the early phase, AR manifests as an IgE-mediated immune response to inhaled allergens, leading to inflammation primarily driven by Th2 cells. Early-phase reactions typically occur within 5-15 minutes after antigen exposure and result in mast cell degranulation. This process leads to the release of preformed and newly synthesized inflammatory mediators, particularly histamine, which plays a central role in the progression of AR [10]. The late-phase response is characterized by a sustained inflammatory state with cellular infiltration, predominantly involving eosinophils, basophils, and T lymphocytes. This infiltration leads to increased secretion of inflammatory mediators, including leukotrienes, kinins, and histamine. Consequently, these mediators contribute to symptom persistence and progression of the late-phase response [2,33] (Figure 1).

Classification of AR

The classification of AR has undergone substantial modifications in recent years, following standardized guidelines. Conventionally, AR was categorized based on allergen exposure into two types: perennial, characterized by year-round symptoms, and seasonal, occurring only during specific times of the year. Epidemiological data report that approximately 40% of cases are perennial, 20% are seasonal, and the remaining 40% exhibit features of both [33,34]. Patients with seasonal or intermittent AR commonly experience symptoms such as watery eyes, rhinorrhea, and sneezing. Common triggers of seasonal AR include pollens, dust, mites, cigarette smoke, perfumes, animal dander, and humidity. In contrast, patients with perennial or chronic AR typically report nasal obstruction, persistent congestion, and postnasal drip [34]. These patients often have a genetic predisposition to AR or a previous history of respiratory conditions such as asthma. Seasonal AR is more common among pediatric patients, whereas adults are more prone to perennial AR [34].

**Figure 2:**
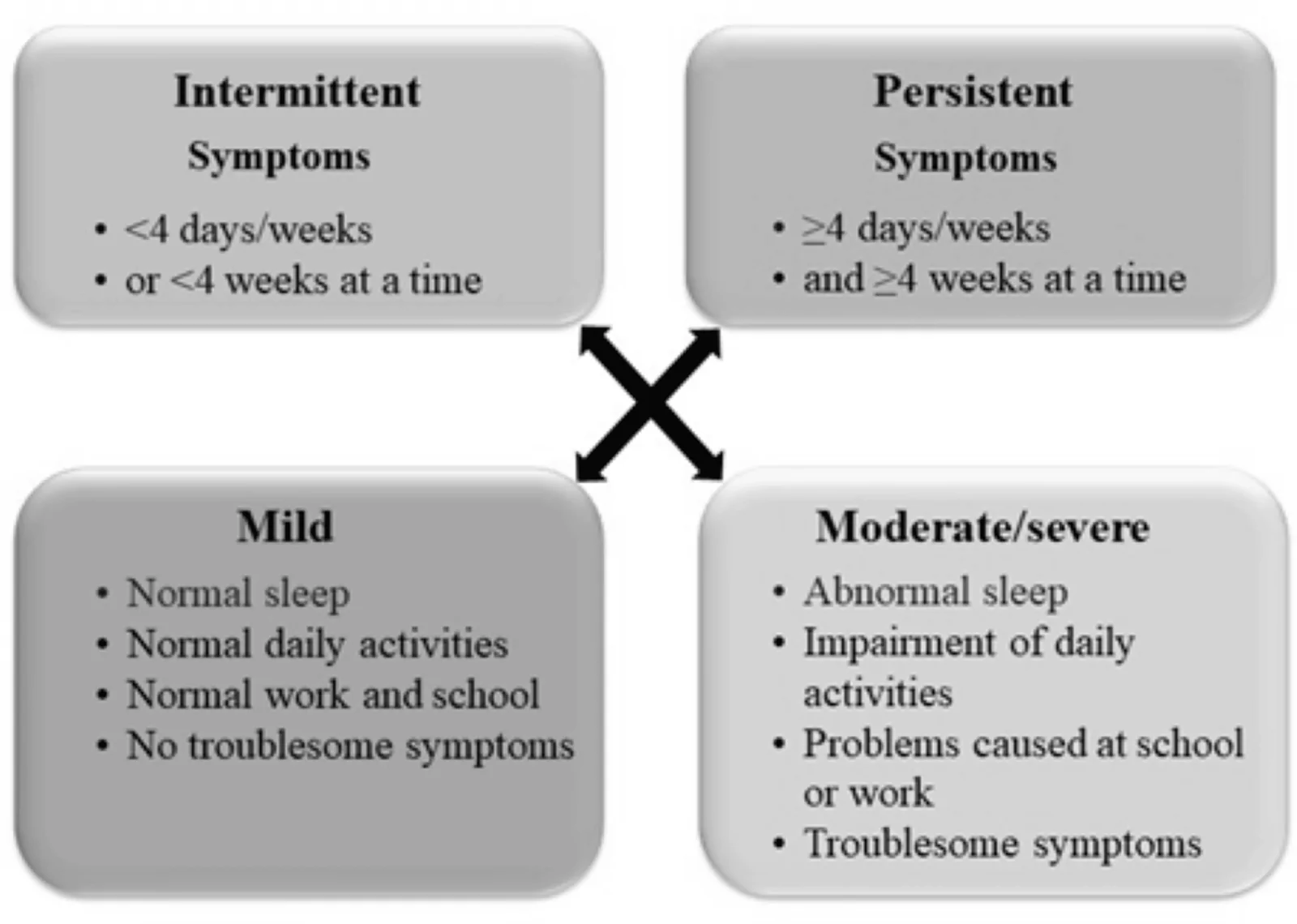
Classification of allergic rhinitis (AR) (ARIA guidelines, 2008) This image was created using Microsoft PowerPoint (Microsoft Corp., Redmond, WA, USA). Source: [30].

The Allergic Rhinitis and its Impact on Asthma (ARIA) classification, introduced in 1999, serves as a valuable framework for distinguishing AR based on the duration and severity of symptoms [30]. It aims to provide a more comprehensive understanding of the condition, enabling improved diagnosis and management strategies. ARIA categorizes AR as intermittent or persistent depending on symptom frequency, measured in days per week and consecutive weeks. The severity is further classified as mild or moderate-to-severe based on patient-reported symptoms and their impact on quality of life (QOL) (Figure 2).

The ARIA classification has been validated with respect to QOL indices and severity scoring, demonstrating its ability to distinguish between different forms of AR based on its impact on daily life and level of severity (mild versus moderate-to-severe) [35].

Key recommendations by global clinical guidelines for the management of AR

Most clinical recommendations for AR are based on the previously conducted RCTs, suggesting allergen immunotherapy for patients with severe and persistent AR, intermittent therapy for those with intermittent symptoms, and long-term continuous or step-wise therapy for individuals with persistent symptoms [22,36-38]. The key features of various global clinical guidelines are summarized below.

The updated 2019 ARIA guidelines emphasize a systematic approach to selecting appropriate AR therapies based on the Grading of Recommendations Assessment, Development, and Evaluation (GRADE) framework. They suggest that combination therapy with intranasal corticosteroids (INCS) and intranasal H1 antihistamines offers greater efficacy and faster symptom relief than INCS monotherapy [1]. Additionally, the guidelines propose that LTRAs may have additive effects when co-administered with antihistamines in the management of AR [4].

The US Clinical Practice Guidelines (2015) recommend oral second-generation antihistamines for patients whose primary symptoms are itching and sneezing [38].

The 2017 Japanese guidelines for AR propose that LTRAs may be comparable to selective antihistamines in alleviating symptoms such as rhinorrhea and sneezing in individuals with mild to moderate nasal congestion [37].

The latest guidelines from the Chinese Society of Allergy (2018) suggest that LTRAs and selective oral antihistamines demonstrate similar efficacy in the management of AR; however, LTRAs may be more effective in alleviating nighttime symptoms [39].

In contrast, the British Society for Allergy and Clinical Immunology (BSACI) guidelines state that the LTRA-antihistamine combination does not provide additional benefit compared to either LTRA or antihistamine monotherapy. They further note that this combination is less effective than topical corticosteroids alone in patients with AR [40].

Conversely, the International Consensus Statement on Allergy and Rhinology-Allergic Rhinitis (ICAR-AR, 2018) supports the use of LTRA-antihistamine combination therapy as a rational approach, particularly in patients with comorbid asthma, inadequate response to antihistamine monotherapy, or intolerance to intranasal corticosteroids [41].

Moreover, the Korean Academy of Asthma, Allergy and Clinical Immunology (KAAACI) guidelines recommend allergen immunotherapy as a preferred strategy for preventing asthma development in patients with AR [36].

Treatment strategies for AR

The management of AR includes three main approaches: allergen avoidance, allergen-specific immunotherapy, and pharmacotherapy.

Allergen Avoidance

It can be used as a non-pharmacological intervention to alleviate or eliminate AR symptoms and reduce the need for ongoing pharmacotherapy. This is a practical approach, particularly when allergens are identified through allergy testing or patient self-reporting. Patients can take appropriate measures to minimize exposure to triggers such as mold, pollen, and animal dander [42].

Allergen-Specific Immunotherapy in AR

Allergen-specific immunotherapy has emerged as a viable and widely used therapeutic approach for the effective management of AR [2]. This treatment is based on the principle of desensitization, in which the immune system is exposed to controlled amounts of allergens, leading to a gradual reduction in the severity of the allergic response over time and providing long-term preventive benefits [43]. Subcutaneous immunotherapy (SCIT) is specifically recommended for patients with severe AR whose symptoms remain inadequately controlled despite ongoing pharmacotherapeutic interventions [2]. This procedure involves the administration of allergens via subcutaneous injections, with a low risk of systemic allergic reactions. Following injection, patients should be monitored for 30-60 minutes [43]. In addition, sublingual immunotherapy (SLIT) may be used for selected allergens such as molds, grass pollen, and pet dander. The frequency of administration may range from three times per week to daily, depending on the prescribed treatment regimen [43]. Sublingual administration is generally considered to have better tolerability than subcutaneous administration; however, the range of allergens currently addressed is limited mainly to specific grass and tree pollens [42].

Pharmacotherapeutic Management of AR

Therapeutic approaches for the management of AR primarily focus on symptom relief using various medications, including oral H1 antihistamines [44], antileukotrienes, oral or intranasal decongestants [3], mast cell stabilizers [45], intranasal corticosteroids [46], and anticholinergics [42] (Table 1).

**Table 1:** A comprehensive overview of pharmacological treatment approaches for allergic rhinitis (AR)

Pharma category	Mechanism of action	Examples
Oral H1 antihistamines (second generation)	Oral H1 antihistamines work by inhibiting the interaction between histamine and H1 receptors, thus preventing histamine-mediated inflammatory responses	Cetirizine, bilastine, desloratadine, fexofenadine, loratadine
Antileukotrienes	Antileukotrienes work by blocking either the action of leukotrienes on cells or their production and help relieve allergic responses and asthma symptoms	Montelukast, pranlukast, zafirlukast
Oral/intranasal decongestants	When bound to α-adrenergic receptors, oral decongestants work by constricting blood vessels in the nasal passages and reducing swelling and congestion	Ephedrine, phenylephrine, phenylpropanolamine, pseudo-ephedrine, oxymetazoline, tetrahydrozoline, xylometazoline
Mast cell stabilizers	By stabilizing mast cells and inhibiting the release of inflammatory mediators, these drugs help prevent or decrease the symptoms associated with conditions driven by mast cell activation, such as allergic reactions and anaphylaxis	Cromolyn
Intranasal corticosteroids	Intranasal corticosteroids affect both initial and delayed inflammatory response through suppression of inflammatory enzymes, delayed hypersensitivity and lymphocyte proliferation	Beclomethasone, budesonide, ciclesonide, fluticasone furoate, fluticasone propionate, mometasone furoate, triamcinolone acetonide
Anticholinergics	Anticholinergics are muscarinic receptor antagonists that block acetylcholine-mediated physiological effects, viz., enhanced mucus secretion, bronchoconstriction, inflammation, and airway remodeling	Ipratropium

Montelukast

**Figure 3:**
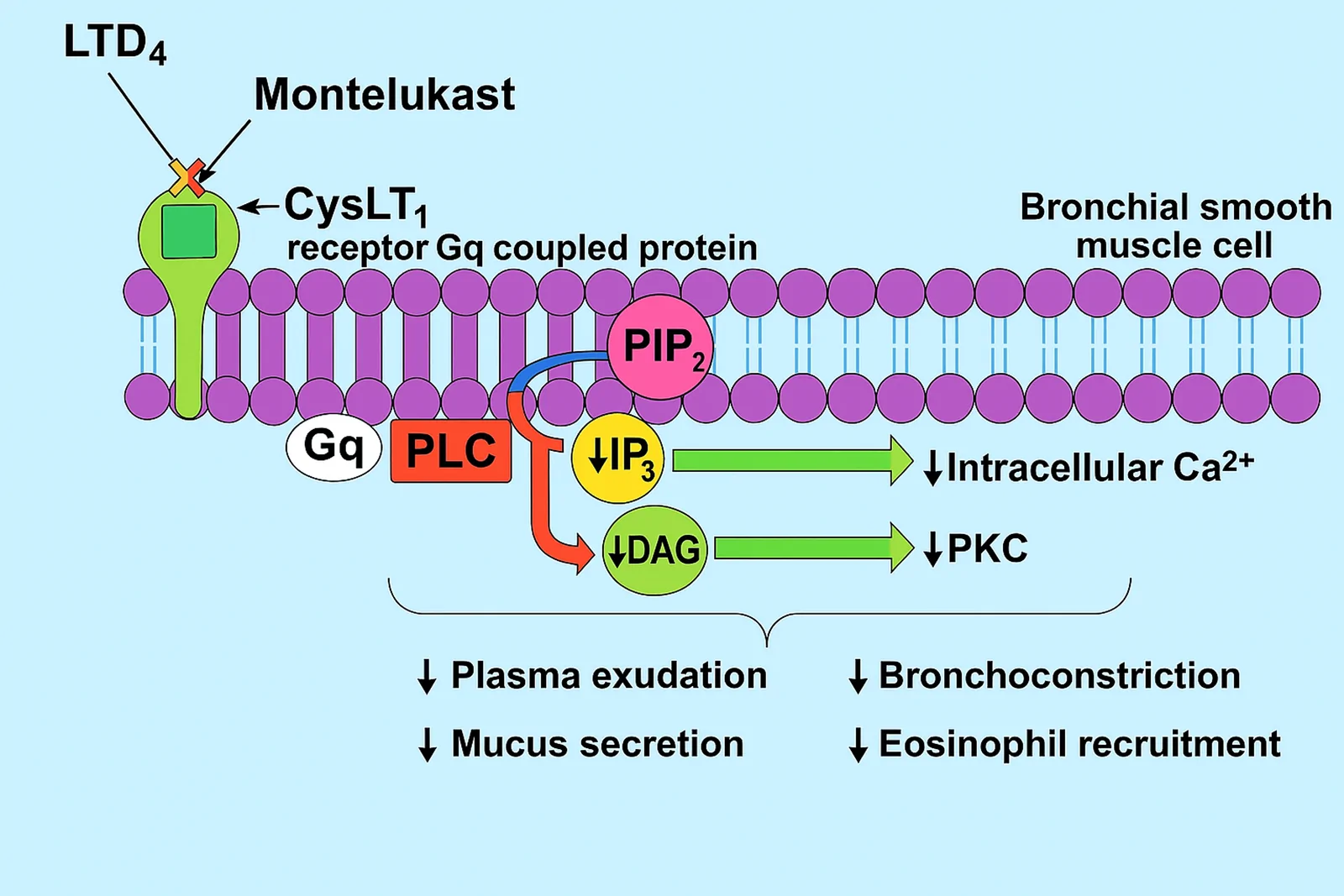
Mechanism of action of montelukast Cells within the immune system produce leukotrienes in response to stimuli, like allergens and infections. Inhibiting LTD4 can prevent inflammatory events, such as migration of neutrophils or eosinophils, bronchoconstriction, and airway edema. Montelukast binds with leukotriene receptors and inhibits leukotriene-mediated effects, namely, impaired cellular activity, smooth muscle contraction, and plasma exudation. PIP_2_: phosphatidylinositol 4,5-bisphosphate, PLC: phospholipase C, IP3: inositol trisphosphate, DAG: diacylglycerol, PKC: protein kinase C. This image was created using Microsoft PowerPoint (Microsoft Corp., Redmond, WA, USA). Source: [47-49].

Montelukast is an LTRA that selectively blocks cysteinyl leukotriene receptor 1 (CysLTR1) and exhibits potent bronchodilatory and anti-inflammatory properties. It acts as a selective, competitive inhibitor of CysLTR1, thereby preventing the binding of leukotriene D4 (LTD4) to the receptor (Figure 3).

LTD4 is a potent inflammatory mediator that can cause a wide range of symptoms, including mucus production, bronchoconstriction, and tissue swelling. LTD4-mediated effects may result in bronchoconstriction in patients with asthma and other respiratory conditions, thereby leading to breathing difficulties [47,48]. At the molecular level, montelukast not only acts as a CysLTR1 antagonist but also inhibits NF-κB activation in various cell types, including endothelial cells, epithelial cells, T cells, and monocytes/macrophages [49]. Table 2 presents the pharmacokinetic (PK) profile of montelukast.

**Table 2:** Pharmacokinetic (PK) profile of montelukast

Population/age group	Dose of montelukast	Maximum plasma concentration (Cmax)	Time to reach Cmax (Tmax)	Notes
Adults	10 mg	Not specified	3-4 hours	Oral bioavailability ≈ 64%; rapid GI absorption
Children ≤8 years	5 mg (chewable tablet)	0.50 mg/L	2.6 hours	Chewable tablet; data from PK study
Children ≤8 years)	4 mg (chewable tablet)	0.47 mg/L	2.6 hours	Chewable tablet; similar exposure to 5 mg
Infants 6-24 months	4 mg (oral)	514 ng/mL	2.2 hours	Oral granules; slightly faster absorption
General safety				Low potential for drug interactions; generally well‑tolerated

Several RCTs have recently been conducted to investigate the therapeutic potential of montelukast in AR, asthma, and COVID-19 infection [50-53]. These studies have shown that it effectively reduces sputum eosinophil levels, exhaled nitric oxide levels, peak flow variability, bronchoconstriction, and bronchial hyper-responsiveness in patients with asthma [54]. Moreover, montelukast administered orally at 10 mg/day for four weeks has been shown to be effective in treating perennial AR in patients with asthma, as evidenced by improvements in lung function parameters [51] and QOL [53]. Treatment guidelines for AR and asthma have also incorporated findings from several studies, supporting the effectiveness of LTRAs, particularly montelukast [20,55,56]. In a crossover study involving 12 asthmatic patients undergoing antigen challenge, montelukast inhibited both early- and late-phase bronchoconstriction. Additionally, studies have shown that short-term treatment with montelukast, either as monotherapy for four weeks or in combination with inhaled corticosteroids (ICS) for three weeks, can improve small airway function and reduce inflammatory markers in both children and adults with asthma [56-58].

Bilastine

**Figure 4:**
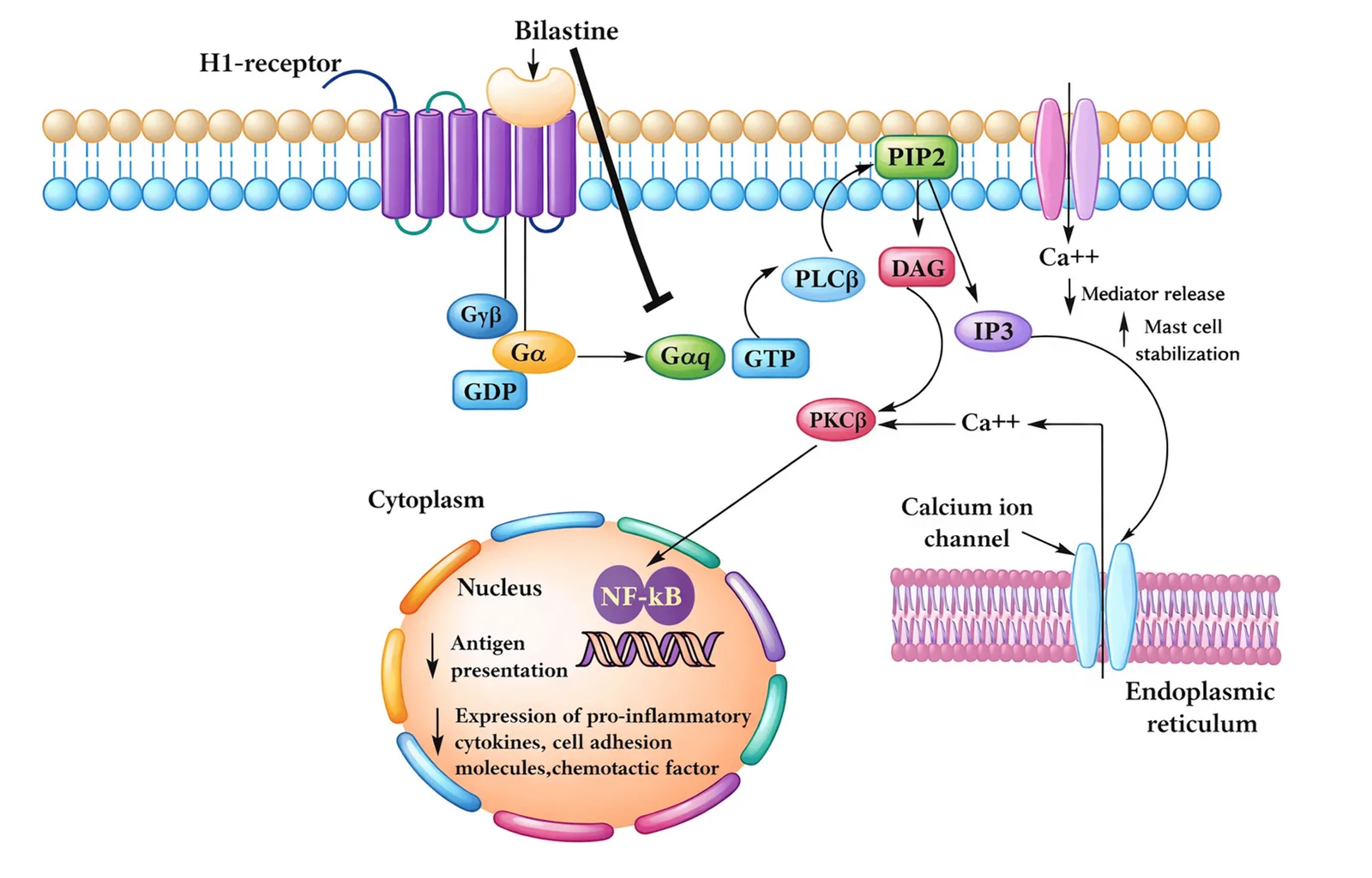
Mechanism of action of bilastine GDP: guanosine-5’-diphosphate, NF-κB: nuclear factor kappa B, DAG: diacylglycerol, PIP_2_: phosphatidylinositol 4,5-bisphosphate, PLC: phospholipase C, PKC: protein kinase C, GTP: guanosine-5’-triphosphate, IP3: inositol triphosphate. Image created using Microsoft PowerPoint (Microsoft Corp., Redmond, WA, USA). Source: [22].

Bilastine is an antihistamine approved for the treatment of both urticaria and AR. As a second-generation, non-sedating antihistamine, it works by blocking histamine H1 receptors, thereby reducing allergic symptoms such as rhinorrhea, sneezing, and itching [59]. It is a selective H1 receptor antagonist that effectively counteracts allergic reactions mediated by sensitized immune cells. These cells produce IgE antibodies that bind to mast cells, leading to their activation and subsequent release of histamine and other inflammatory mediators. This cascade of events gives rise to the symptoms associated with AR. Bilastine binds to H1 receptors and inhibits histamine-mediated effects, thereby reducing mast cell activation and subsequent histamine release, which ultimately alleviates AR symptoms in patients [22] (Figure 4). Table 3 presents the PK profile of bilastine.

**Table 3:** Pharmacokinetic (PK) profile of bilastine

Parameter	Value/description
Standard adult dose	20 mg once daily
Route of administration	Oral
Absorption	Rapid absorption
Time to reach Cmax (Tmax)	1-1.5 hours
Oral bioavailability	~60%
Plasma protein binding	84%-90%
Metabolism	Does not undergo significant hepatic metabolism
Excretion	~90% excreted unchanged
Urinary excretion	33%
Fecal excretion	67%
Elimination half-life (t½)	12-14.5 hours
Tolerability	Generally well tolerated
Comparative effect	Shows better reduction in itching than rupatadine and desloratadine
Dose adjustment	Not required in elderly or in patients with renal or hepatic impairment

Numerous studies have demonstrated the broad application of bilastine in the treatment of AR [37,60,61]. In an earlier randomized trial, bilastine was found to be more efficacious than fexofenadine in treating perennial AR, suggesting its therapeutic potential in chronic spontaneous urticaria that is unresponsive to other antihistamines, including fexofenadine [37]. In a comparative study of bilastine (20 mg), cetirizine (10 mg), and placebo, bilastine was found to be more effective than placebo and demonstrated similar efficacy to cetirizine in reducing symptoms associated with seasonal AR. Additionally, bilastine exhibited fewer adverse effects than cetirizine [62]. The effectiveness of bilastine at a daily dose of 20 mg has also been evaluated, showing significant improvement in both nasal and non-nasal allergic symptoms. Bilastine demonstrated a notable reduction in overall nasal symptom scores compared with both placebo and fexofenadine in patients with perennial AR [37].

Clinical effects of montelukast (10 mg) and bilastine (40 mg)

Several RCTs are currently being conducted to examine the combined effects of montelukast (10 mg) and bilastine (20 mg) [63,64]. Nevertheless, fluctuations in environmental allergen levels can intensify allergic reactions, thereby necessitating dose adjustments of certain medications to manage exacerbated symptoms [65]. The clinical effects of montelukast (10 mg) have demonstrated significant improvement in nasal and ocular symptoms in patients with AR after a two-week treatment period compared with placebo. Van Adelsberg et al. conducted a study to investigate the efficacy and tolerability of montelukast 10 mg in adolescents and adults with seasonal AR. The findings indicated that montelukast showed superior efficacy in improving daytime nasal symptom scores and other key outcomes, including nighttime symptoms, daytime ocular symptoms, and overall QOL, compared with placebo [66]. Additionally, in a study involving 20 healthy volunteers, a double dose of bilastine (40 mg) did not adversely affect psychomotor performance, as assessed using objective tests such as the fine motor test (motor activity), the critical flicker-fusion test (perception), the simple reaction time test (associative integration), and the d2 cancellation test (attention) [67]. In a double-blind, crossover study involving 22 healthy volunteers, the effects of bilastine (20 mg and 40 mg) on driving performance were assessed using a standardized road driving test. Participants were randomly assigned to receive bilastine 20 mg or 40 mg, 50 mg of an active control, or placebo once daily for eight days. The results showed no significant differences in car weaving between the 20 mg and 40 mg doses of bilastine on both day 1 and day 8. Furthermore, bilastine, after single or repeated dosing, did not negatively affect driving performance, confirming its safety for use in traffic, even at doses up to 40 mg [68]. Notably, the incidence of adverse events (AEs) with bilastine 40 mg was comparable to placebo, indicating good tolerability even at doses higher than the standard daily dose [69].

Current scenario on FDC of montelukast and bilastine

Clinical investigations support the combined use of montelukast and antihistamines for effectively alleviating AR symptoms [70]. A survey on practice patterns related to the diagnosis and management of AR in India reported that the montelukast-antihistamine combination is preferred by approximately 51.6% of physicians for treating patients with mild to severe AR, including those with mild asthma or those who do not respond adequately to antihistamine monotherapy [71]. This therapeutic combination potentially targets two key pathways involved in AR pathophysiology, namely, leukotrienes, which are responsible for increased vascular permeability and nasal airway resistance, and histamine, which contributes to acute allergic symptoms [72]. Specifically, the combined use of montelukast and bilastine exhibits a synergistic effect in improving nasal symptoms by inhibiting both early- and late-phase allergic responses involved in AR pathophysiology [66]. Growing evidence supports the effectiveness of montelukast-bilastine combination therapy as a more effective approach for managing moderate-to-severe persistent AR compared with monotherapy [66,73]. A comparative analysis investigating LTRA plus oral H1 antihistamine therapy versus H1 antihistamine monotherapy in AR management demonstrated significant improvements in daytime and composite nasal symptoms, including itching, sneezing, and rhinorrhea, with the combination therapy. However, ocular symptoms and nighttime nasal symptoms were not significantly improved with LTRA plus oral H1 antihistamine therapy [74]. CysLT1 and histamine are key inflammatory mediators involved in the progression of both asthma and seasonal AR [75]. Recently, a randomized, double-blind, comparative, parallel-group study evaluated the efficacy and safety of a FDC of montelukast (10 mg) and bilastine (20 mg) administered once daily for four weeks in patients with AR. The study reported statistically significant improvements in AR-related discomfort, as measured by the Clinical Global Impression (CGI) and visual analog scale (VAS) scores, compared with placebo. Moreover, the combination was well tolerated and effective in relieving AR symptoms [63]. In a similar study, the montelukast-bilastine combination demonstrated faster symptom relief and reduced recurrence, suggesting superior efficacy in managing AR symptoms compared with the montelukast-levocetirizine combination. Additionally, it showed improvements in sleep quality, with a favorable safety profile characterized by minimal long-term side effects and fewer dose adjustments compared with the montelukast-levocetirizine combination [64]. A list of approved FDCs containing montelukast and bilastine for the treatment of AR in India is provided in Table 4.

**Table 4:** Approved fixed-dose combination (FDCs) of montelukast and bilastine for AR management in India

Doses	Dosage forms	Administration route	Approval year
Montelukast (10 mg) + bilastine (40 mg)	Film-coated tablets	Oral	2024
Montelukast (10 mg) + bilastine (20 mg)	Orodispersible tablets	Oral	2022
Montelukast (4 mg) + bilastine (10 mg)	Suspension	Oral	2022
Montelukast (10 mg) + bilastine (20 mg)	Tablet	Oral	2020
Montelukast (10 mg) + bilastine (20 mg)	Bilayered tablets	Oral	2020

ODT formulations are rapidly disintegrating tablets with adequate hardness, rapid disintegration in the oral cavity, and an enhanced dissolution rate. ODTs improve the bioavailability and efficacy of drugs by facilitating rapid and efficient absorption from the pregastric regions of the gastrointestinal tract [76]. The use of ODT formulations also improves patient compliance, making them an effective option for individuals who experience difficulty swallowing conventional oral tablets [76,77]. Montelukast ODT has been studied in patients with AR and mild-to-moderate asthma. Study findings revealed a high satisfaction rate (98.5%) after administration for less than four weeks in adults with AR or asthma, particularly in terms of tablet size, taste, disintegration speed, and overall ease of use, as measured by the Treatment Satisfaction Questionnaire for Medication-9 (TSQM-9) [78]. Additionally, in a pediatric safety trial, administering bilastine ODT dissolved in a small amount of water to toddlers resulted in high treatment compliance (98.5%), comparable to placebo (98.6%). The study suggested that bilastine ODT may be a convenient therapeutic option for children [68]. ODT formulations of montelukast and bilastine may provide comprehensive relief from allergic symptoms by targeting multiple pathways involved in allergic reactions, including itching, sneezing, and nasal congestion. Moreover, both drugs exhibit a favorable safety profile with fewer adverse effects, making them a rational choice for long-term management of chronic AR [77]. However, these findings are largely limited to patient satisfaction outcomes, and satisfaction alone should not be used as the sole measure of treatment effectiveness. Therefore, comprehensive studies assessing multiple clinical endpoints are needed to fully evaluate the effectiveness and benefits of montelukast and bilastine ODTs. With growing interest, several studies are currently underway (CTRI/2022/03/040760, CTRI/2023/10/058571, CTRI/2019/10/021567) to evaluate the clinical effectiveness of combined montelukast and bilastine ODT therapy for AR in both adults and children in India.

Safety

The safety profile of the combination therapy comprising montelukast and bilastine has been evaluated in several clinical studies assessing its tolerability and safety. A phase III randomized, double-blind, multicenter clinical trial conducted in patients with AR demonstrated that the FDC of montelukast (10 mg) and bilastine (20 mg) was generally safe and well tolerated [63]. In this study, the incidence of AEs was low, and most reported events were mild to moderate in severity. Comparative studies evaluating the safety of the montelukast-bilastine combination against other antihistamine-based regimens, such as montelukast with levocetirizine, have also suggested that the bilastine-based regimen may have a more favorable tolerability profile, particularly due to the minimal sedative effects associated with bilastine [79].

Although this combination is generally considered safe, certain safety considerations remain relevant. Montelukast has been associated with rare neuropsychiatric adverse effects, including sleep disturbances, mood changes, and anxiety; therefore, clinical monitoring is recommended, especially during long-term therapy [19]. Overall, current literature indicates that the montelukast-bilastine combination offers a favorable balance between efficacy and safety, making it a well-tolerated therapeutic option.

Discussion

This review significantly advances the understanding of AR treatment using the combination of montelukast and bilastine. The montelukast-bilastine approach integrates the leukotriene inhibitory action of montelukast with the antihistaminic effects of bilastine, a second-generation antihistamine known for its favorable efficacy and safety profile [64].

Montelukast offers a rational approach to controlling AR-associated inflammatory symptoms, and its effectiveness may be further enhanced when used in combination with an antihistamine in clinical settings, thereby providing improved symptomatic relief. The combination therapy acts on multiple inflammatory pathways, potentially enhancing the overall clinical efficacy of both agents [59]. Notably, montelukast-bilastine therapy exerts effects on both the early and late phases of allergic reactions in patients with AR [66]. Moreover, expert opinion suggests that the montelukast-bilastine combination may be an effective treatment strategy for individuals with hyperreactive airway disease, particularly those with asthma, to achieve better clinical outcomes [66]. A recent study demonstrated that montelukast (10 mg) and bilastine (20 mg) had similar efficacy to the montelukast-levocetirizine combination in reducing symptoms and improving QOL in patients with AR over a four-week treatment period, as assessed by patient-reported discomfort, individual symptom scores, and total severity scores [63]. Montelukast (10 mg) has also been shown to significantly improve nasal and ocular symptoms in patients with AR after a two-week treatment period, providing relief from allergic symptoms and, in some cases, asthma-related manifestations in patients with comorbid conditions. Additionally, the non-sedative profile of bilastine has been supported by studies evaluating higher doses, including driving simulation and on-road driving tests, which demonstrated no impairment in psychomotor or driving performance [68]. The clinical effects of multiple doses of bilastine (20 mg, 40 mg, and 80 mg) administered once daily for seven days have also shown a significant reduction in the critical temperature threshold in patients with cold contact urticaria, without causing sedation even at higher doses [68]. However, due to limited available evidence, further research is needed to evaluate the efficacy and safety of montelukast and bilastine across diverse patient populations, including elderly individuals and children. Additional studies are also warranted in patients with seasonal allergic rhinoconjunctivitis, particularly since the study by Lavorini et al. did not demonstrate a significant impact on efficacy outcomes with the montelukast-bilastine combination [75]. These evaluations should include both short-term and long-term treatment periods.

## Conclusions

Growing evidence supports the use of montelukast-bilastine FDCs as an effective therapeutic approach for the management of moderate-to-severe AR compared with monotherapy. However, most studies conducted to date have focused on short-term outcomes, with limited data available on the long-term safety and efficacy of this combination in AR management. Therefore, further research is warranted to evaluate the long-term benefits, potential adverse effects, and durability of treatment response associated with these medications. Given that the severity and clinical presentation of AR can vary significantly, additional studies are needed to assess the clinical efficacy of montelukast and bilastine across diverse patient populations, including elderly individuals and children.
